# 4-(Cyclo­hexyl­sulfan­yl)-1-[(*E*)-2-(cyclo­hexyl­sulfan­yl)-1-phenyl­ethen­yl]-3-phenyl-1*H*-pyrazole

**DOI:** 10.1107/S1600536808033126

**Published:** 2008-10-18

**Authors:** P. Ramesh, A. Subbiahpandi, Ramaiyan Manikannan, S. Muthusubramanian, M. N. Ponnuswamy

**Affiliations:** a Department of Physics, Presidency College (Autonomous), Chennai 600 005, India; b Department of Organic Chemistry, School of Chemistry, Madurai Kamaraj University, Madurai 625 021, India; c Centre of Advanced Study in Crystallography and Biophysics, University of Madras, Guindy Campus, Chennai 600 025, India

## Abstract

In the title compound, C_29_H_34_N_2_S_2_, the pyrazole ring is planar and both cyclo­hexane rings adopt chair conformations. The dihedral angles between the pyrazole ring and the two benzene rings are 59.9 (2) and 19.8 (2)°. The conformation and packing of the mol­ecules in the unit cell are stabilized by a weak intra­molecular C—H⋯S and C—H⋯N interactions, in addition to van der Waals forces.

## Related literature

For pharmacological and medicinal properties of pyrazole derivatives, see: Baraldi *et al.* (1998 ▶); Bruno *et al.* (1990 ▶); Cottineau *et al.* (2002 ▶); Londershausen (1996 ▶); Chen & Li (1998 ▶); Mishra *et al.* (1998 ▶); Smith *et al.* (2001 ▶). For hybridization, see: Beddoes *et al.* (1986 ▶). For puckering and asymmetry analysis, see: Cremer & Pople (1975 ▶); Nardelli (1983 ▶). Manikannan (2008 ▶) describes other compounds formed along with the title compound in its synthesis.
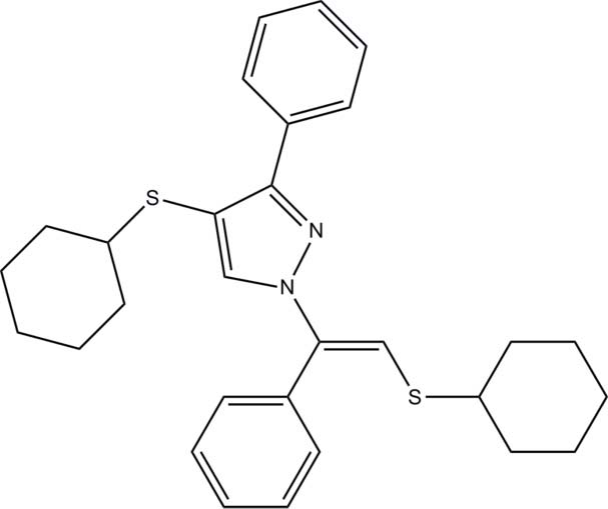


## Experimental

### 

#### Crystal data

C_29_H_34_N_2_S_2_
*M* *_r_* = 474.70
Orthorhombic, 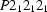

*a* = 6.3859 (5) Å
*b* = 19.1596 (17) Å
*c* = 21.337 (2) Å
*V* = 2610.7 (4) Å^3^
*Z* = 4
Mo *K*α radiation
μ = 0.22 mm^−1^
*T* = 293 (2) K
0.25 × 0.21 × 0.19 mm

#### Data collection

Bruker APEXII CCD area-detector diffractometer
Absorption correction: multi-scan (*SADABS*; Sheldrick, 2001 ▶) *T*_min_ = 0.936, *T*_max_ = 0.965
19948 measured reflections
7787 independent reflections
4945 reflections with *I* > 2σ(*I*)
*R*_int_ = 0.031

#### Refinement

*R*[*F* ^2^ > 2σ(*F* ^2^)] = 0.051
*wR*(*F* ^2^) = 0.149
*S* = 1.02
7787 reflections
298 parameters
H-atom parameters constrained
Δρ_max_ = 0.31 e Å^−3^
Δρ_min_ = −0.17 e Å^−3^
Absolute structure: Flack (1983 ▶), 3381 Friedel pairs
Flack parameter: −0.01 (8)

### 

Data collection: *APEX2* (Bruker, 2004 ▶); cell refinement: *APEX2*; data reduction: *SAINT* (Bruker, 2004 ▶); program(s) used to solve structure: *SHELXS97* (Sheldrick, 2008 ▶); program(s) used to refine structure: *SHELXL97* (Sheldrick, 2008 ▶); molecular graphics: *ORTEP-3* (Farrugia, 1997 ▶); software used to prepare material for publication: *SHELXL97* and *PLATON* (Spek, 2003 ▶).

## Supplementary Material

Crystal structure: contains datablocks global, I. DOI: 10.1107/S1600536808033126/zl2138sup1.cif

Structure factors: contains datablocks I. DOI: 10.1107/S1600536808033126/zl2138Isup2.hkl

Additional supplementary materials:  crystallographic information; 3D view; checkCIF report

## Figures and Tables

**Table 1:** Hydrogen-bond geometry (Å, °)

*D*—H⋯*A*	*D*—H	H⋯*A*	*D*⋯*A*	*D*—H⋯*A*
C7—H7⋯N1	0.93	2.40	2.760 (4)	103
C27—H27⋯S2	0.93	2.80	3.450 (4)	128
C31—H31⋯N1	0.93	2.46	2.786 (4)	101

**Fig. 1.**
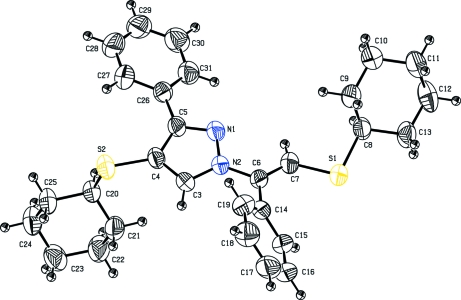
Perspective view of the molecule with thermal ellipsoids drawn at the 50% probability level. H atoms are shown as small circles of arbitrary radius.

**Fig. 2.**
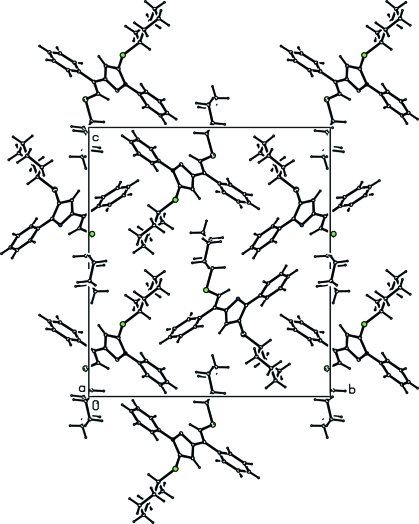
The packing of the molecules in the unit cell viewed down the a axis.

## supplementary crystallographic information

### Comment

Pyrazole derivatives possess significant antiarrhythmic and sedative (Bruno *et al.*, 1990), hypoglycemic (Cottineau *et al.*, 2002), antiviral (Baraldi *et al.*, 1998), and pesticidal (Londershausen, 1996) properties. Some pyrazole derivatives were successfully tested for their antifungal (Chen & Li, 1998), antihistaminic (Mishra *et al.*, 1998) and anti-inflammatory  (Smith *et al.*, 2001) properties.

An *ORTEP* plot of the molecule is shown in Fig. 1 and a packing plot in Fig. 2. The pyrazole ring adopts a planar conformation. The sum of the angles at N1 of the pyrazole ring (359.95°) is in accordance with *sp*^2^ hybridization (Beddoes *et al.*, 1986). Both the cyclohexane rings in the molecule adopt chair conformations which can be seen from the puckering (Cremer & Pople, 1975) and the asymmetry parameters (Nardelli, 1983). The values for the ring C8-C13 are: q_2_ = 0.010 (4) Å, q_3_ = - 0.562 (4) Å, π = 186 (22)°, Δs(C9) and Δs(C12) =0.5 (4)° and for ring C20-C25 are: q_2_ = 0.014 (4) Å, q_3_ = - 0.572 (4) Å, π = 132 (14)°, Δs(C22) and Δs(C25) = 0.3 (3)°.

The best least-squares planes calculated for the two cyclohexane rings (atoms C8, C9, C11 & C12 lie in the plane and C10 & C13 deviate for one of the rings; atoms C21, C22, C24 & C25 lie in the plane and C20 & C23 deviate for the other ring ) are twisted from the pyrazole ring by 50.06 (17)° and 69.71 (15), respectively. The crystal packing is augmented by weak intramolecular C—H···N and C—H···S interactions in addition to van der Waals forces.

### Experimental

A mixture of 2-(cyclohexylsulfanyl)-1-phenyl-1-ethanone N-[(E)-2- (cyclohexylsulfanyl)-1-phenylethylidene]hydrazone (0.003 mole) and 3 ml of dimethyl formamide were kept in an ice bath at 273 K and phosphorus oxychloride (0.024 mole) was added dropwise for 5 to 10 minutes. The reaction mixture was then kept in a microwave oven at 600 W for 30–60 sec. The process of the reaction was monitored by TLC. After completion of the reaction, the reaction mixture was poured into crushed ice and extracted with dichloromethane. The organic layer was dried with anhydrous sodium sulfate. The different compounds present in the mixture were separated by column chromatography using petroleum ether and ethyl acetate as the eluent (99:1 v/v, R_f_ index of the title compound: 0.8336). The isolated title compound was recrystallized from dichloromethane to obtain 4-(cyclohexylsulfanyl) -1-[(*E*)-2-(cyclohexylsulfanyl)-1-phenylethenyl]-3 -phenyl-1*H *-pyrazole (title compound) and 4-(cyclohexyl sulfanyl)-1-[*Z*-2 -(cyclohexylsulfanyl)-1-phenyl-1-ethenyl]-3-phenyl-1*H*-pyrazole in 38% and 60% yield. The compounds identified through column are characterized by NMR studies (Manikannan, 2008).

### Refinement

H atoms were positioned geometrically (C—H = 0.93–0.98 Å) and allowed to ride on their parent atoms, with *U*_iso_(H) = 1.2*U*_eq_(C) for all H atoms.

### Crystal data

**Table:**

C_29_H_34_N_2_S_2_	*F*(000) = 1016
*M**_r_* = 474.70	*D*_x_ = 1.208 Mg m^−^^3^
Orthorhombic, *P*2_1_2_1_2_1_	Mo *K*α radiation, λ = 0.71073 Å
Hall symbol: P 2ac 2ab	Cell parameters from 2320 reflections
*a* = 6.3859 (5) Å	θ = 1.4–30.3°
*b* = 19.1596 (17) Å	µ = 0.22 mm^−^^1^
*c* = 21.337 (2) Å	*T* = 293 K
*V* = 2610.7 (4) Å^3^	Block, colorless
*Z* = 4	0.25 × 0.21 × 0.19 mm

### Data collection

**Table:**

Bruker APEXII CCD area-detector diffractometer	7787 independent reflections
Radiation source: fine-focus sealed tube	4945 reflections with *I* > 2σ(*I*)
graphite	*R*_int_ = 0.031
ω and φ scans	θ_max_ = 30.3°, θ_min_ = 1.4°
Absorption correction: multi-scan (SADABS; Sheldrick, 2001)	*h* = −9→8
*T*_min_ = 0.936, *T*_max_ = 0.965	*k* = −27→27
19948 measured reflections	*l* = −30→21

### Refinement

**Table:**

Refinement on *F*^2^	Secondary atom site location: difference Fourier map
Least-squares matrix: full	Hydrogen site location: inferred from neighbouring sites
*R*[*F*^2^ > 2σ(*F*^2^)] = 0.051	H-atom parameters constrained
*wR*(*F*^2^) = 0.149	*w* = 1/[σ^2^(*F*_o_^2^) + (0.0724*P*)^2^ + 0.1893*P*] where *P* = (*F*_o_^2^ + 2*F*_c_^2^)/3
*S* = 1.02	(Δ/σ)_max_ < 0.001
7787 reflections	Δρ_max_ = 0.31 e Å^−^^3^
298 parameters	Δρ_min_ = −0.17 e Å^−^^3^
0 restraints	Absolute structure: Flack (1983), 3381 Friedel pairs
Primary atom site location: structure-invariant direct methods	Flack parameter: −0.01 (8)

### Special details

**Table:**

Geometry. All e.s.d.'s (except the e.s.d. in the dihedral angle between two l.s. planes) are estimated using the full covariance matrix. The cell e.s.d.'s are taken into account individually in the estimation of e.s.d.'s in distances, angles and torsion angles; correlations between e.s.d.'s in cell parameters are only used when they are defined by crystal symmetry. An approximate (isotropic) treatment of cell e.s.d.'s is used for estimating e.s.d.'s involving l.s. planes.
Refinement. Refinement of *F*^2^ against ALL reflections. The weighted *R*-factor *wR* and goodness of fit *S* are based on *F*^2^, conventional *R*-factors *R* are based on *F*, with *F* set to zero for negative *F*^2^. The threshold expression of *F*^2^ > σ(*F*^2^) is used only for calculating *R*-factors(gt) *etc*. and is not relevant to the choice of reflections for refinement. *R*-factors based on *F*^2^ are statistically about twice as large as those based on *F*, and *R*- factors based on ALL data will be even larger.

### Fractional atomic coordinates and isotropic or equivalent isotropic displacement parameters (Å^2^)

**Table:**

	*x*	*y*	*z*	*U*_iso_*/*U*_eq_	
S1	1.40978 (12)	0.48628 (4)	0.39564 (3)	0.0608 (2)	
S2	0.40247 (11)	0.64092 (3)	0.23133 (3)	0.05671 (18)	
N1	0.8399 (3)	0.61798 (10)	0.36277 (10)	0.0481 (5)	
N2	0.8853 (3)	0.56795 (10)	0.32015 (9)	0.0467 (4)	
C3	0.7544 (4)	0.57046 (13)	0.27104 (12)	0.0490 (5)	
H3	0.7592	0.5416	0.2360	0.059*	
C4	0.6130 (4)	0.62265 (12)	0.28137 (11)	0.0480 (5)	
C5	0.6735 (4)	0.65155 (12)	0.33978 (11)	0.0466 (5)	
C6	1.0582 (4)	0.52201 (12)	0.33064 (11)	0.0450 (5)	
C7	1.1971 (4)	0.53826 (14)	0.37473 (12)	0.0532 (6)	
H7	1.1795	0.5803	0.3959	0.064*	
C8	1.4367 (4)	0.51105 (14)	0.47716 (13)	0.0548 (6)	
H8	1.4470	0.5620	0.4798	0.066*	
C9	1.2542 (5)	0.4872 (2)	0.51640 (15)	0.0790 (10)	
H9A	1.2377	0.4372	0.5117	0.095*	
H9B	1.1275	0.5093	0.5011	0.095*	
C10	1.2808 (6)	0.5042 (3)	0.58490 (18)	0.1023 (14)	
H10A	1.1638	0.4854	0.6085	0.123*	
H10B	1.2823	0.5544	0.5906	0.123*	
C11	1.4836 (5)	0.4733 (2)	0.60921 (17)	0.0874 (10)	
H11A	1.5042	0.4877	0.6524	0.105*	
H11B	1.4741	0.4228	0.6084	0.105*	
C12	1.6674 (5)	0.49605 (19)	0.57090 (18)	0.0821 (10)	
H12A	1.7925	0.4729	0.5862	0.098*	
H12B	1.6870	0.5460	0.5758	0.098*	
C13	1.6387 (4)	0.47926 (18)	0.50208 (15)	0.0691 (8)	
H13A	1.6350	0.4290	0.4965	0.083*	
H13B	1.7566	0.4974	0.4785	0.083*	
C14	1.0638 (4)	0.45828 (13)	0.29141 (11)	0.0477 (5)	
C15	1.2456 (4)	0.44122 (15)	0.25948 (13)	0.0584 (7)	
H15	1.3617	0.4705	0.2614	0.070*	
C16	1.2534 (5)	0.38010 (18)	0.22451 (14)	0.0707 (8)	
H16	1.3757	0.3683	0.2033	0.085*	
C17	1.0840 (6)	0.33739 (16)	0.22097 (15)	0.0765 (9)	
H17	1.0904	0.2965	0.1975	0.092*	
C18	0.9053 (6)	0.35457 (15)	0.25173 (15)	0.0728 (8)	
H18	0.7890	0.3255	0.2488	0.087*	
C19	0.8942 (5)	0.41443 (14)	0.28721 (13)	0.0589 (6)	
H19	0.7713	0.4252	0.3085	0.071*	
C20	0.5010 (4)	0.71168 (13)	0.18284 (13)	0.0502 (6)	
H20	0.5267	0.7524	0.2096	0.060*	
C21	0.6984 (5)	0.69469 (18)	0.14829 (16)	0.0715 (9)	
H21A	0.6779	0.6526	0.1238	0.086*	
H21B	0.8100	0.6860	0.1781	0.086*	
C22	0.7614 (5)	0.7546 (2)	0.10494 (18)	0.0823 (10)	
H22A	0.7965	0.7953	0.1299	0.099*	
H22B	0.8850	0.7413	0.0814	0.099*	
C23	0.5874 (6)	0.7732 (2)	0.05995 (16)	0.0823 (9)	
H23A	0.6285	0.8133	0.0351	0.099*	
H23B	0.5630	0.7344	0.0316	0.099*	
C24	0.3890 (5)	0.78953 (16)	0.09496 (17)	0.0755 (9)	
H24A	0.2776	0.7990	0.0653	0.091*	
H24B	0.4097	0.8310	0.1202	0.091*	
C25	0.3260 (4)	0.72955 (15)	0.13670 (15)	0.0613 (7)	
H25A	0.2947	0.6890	0.1112	0.074*	
H25B	0.2004	0.7419	0.1597	0.074*	
C26	0.5914 (4)	0.71108 (11)	0.37568 (11)	0.0478 (5)	
C27	0.3886 (5)	0.73724 (14)	0.36781 (15)	0.0626 (7)	
H27	0.2979	0.7165	0.3392	0.075*	
C28	0.3239 (5)	0.79383 (15)	0.40263 (17)	0.0736 (9)	
H28	0.1905	0.8119	0.3961	0.088*	
C29	0.4492 (6)	0.82374 (16)	0.44595 (16)	0.0739 (9)	
H29	0.4020	0.8617	0.4692	0.089*	
C30	0.6466 (6)	0.79767 (16)	0.45541 (16)	0.0755 (9)	
H30	0.7335	0.8175	0.4855	0.091*	
C31	0.7160 (5)	0.74201 (14)	0.42013 (14)	0.0625 (7)	
H31	0.8506	0.7250	0.4266	0.075*	

### Atomic displacement parameters (Å^2^)

**Table:**

	*U*^11^	*U*^22^	*U*^33^	*U*^12^	*U*^13^	*U*^23^
S1	0.0537 (3)	0.0746 (4)	0.0542 (4)	0.0223 (3)	−0.0076 (3)	−0.0031 (3)
S2	0.0476 (3)	0.0660 (4)	0.0565 (4)	−0.0031 (3)	−0.0134 (3)	0.0156 (3)
N1	0.0514 (11)	0.0490 (10)	0.0439 (11)	0.0077 (8)	−0.0057 (9)	0.0028 (9)
N2	0.0481 (11)	0.0523 (10)	0.0396 (10)	0.0112 (9)	−0.0039 (9)	0.0015 (8)
C3	0.0512 (13)	0.0583 (13)	0.0375 (12)	0.0060 (11)	−0.0024 (11)	0.0043 (11)
C4	0.0468 (12)	0.0525 (12)	0.0446 (13)	0.0036 (10)	−0.0073 (11)	0.0103 (10)
C5	0.0483 (12)	0.0474 (12)	0.0440 (13)	0.0046 (10)	−0.0028 (10)	0.0095 (10)
C6	0.0436 (12)	0.0500 (12)	0.0413 (12)	0.0083 (10)	0.0026 (10)	0.0077 (10)
C7	0.0522 (14)	0.0550 (13)	0.0524 (15)	0.0113 (11)	−0.0070 (12)	0.0019 (12)
C8	0.0525 (14)	0.0580 (13)	0.0539 (14)	0.0098 (11)	−0.0140 (12)	−0.0015 (12)
C9	0.0445 (14)	0.131 (3)	0.0620 (19)	0.0160 (17)	−0.0067 (13)	−0.0031 (19)
C10	0.076 (2)	0.172 (4)	0.059 (2)	0.041 (3)	−0.0054 (18)	0.001 (2)
C11	0.073 (2)	0.124 (3)	0.065 (2)	0.014 (2)	−0.0145 (18)	0.017 (2)
C12	0.0695 (19)	0.091 (2)	0.085 (2)	−0.0139 (17)	−0.0374 (18)	0.0172 (19)
C13	0.0418 (15)	0.091 (2)	0.075 (2)	−0.0011 (13)	−0.0132 (13)	0.0102 (17)
C14	0.0514 (13)	0.0534 (12)	0.0383 (12)	0.0087 (11)	0.0007 (10)	0.0052 (10)
C15	0.0529 (14)	0.0738 (17)	0.0485 (15)	0.0122 (12)	0.0076 (12)	0.0020 (13)
C16	0.0710 (18)	0.089 (2)	0.0520 (17)	0.0305 (17)	0.0058 (15)	−0.0081 (15)
C17	0.095 (2)	0.0715 (18)	0.0629 (19)	0.0181 (19)	−0.008 (2)	−0.0199 (15)
C18	0.078 (2)	0.0649 (17)	0.076 (2)	−0.0043 (16)	−0.0062 (19)	−0.0089 (15)
C19	0.0571 (14)	0.0597 (14)	0.0598 (16)	0.0037 (13)	0.0053 (14)	−0.0028 (12)
C20	0.0465 (12)	0.0551 (13)	0.0489 (14)	0.0034 (10)	−0.0100 (11)	0.0050 (11)
C21	0.0566 (16)	0.094 (2)	0.0641 (19)	0.0183 (15)	0.0000 (15)	0.0214 (17)
C22	0.0532 (16)	0.115 (3)	0.079 (2)	0.0054 (17)	0.0047 (16)	0.034 (2)
C23	0.082 (2)	0.101 (2)	0.0641 (19)	0.003 (2)	−0.0034 (19)	0.0330 (17)
C24	0.0654 (18)	0.0767 (19)	0.084 (2)	0.0102 (16)	−0.0185 (18)	0.0206 (16)
C25	0.0490 (14)	0.0675 (16)	0.0673 (18)	0.0021 (12)	−0.0159 (13)	0.0154 (14)
C26	0.0527 (13)	0.0439 (11)	0.0469 (13)	0.0055 (11)	0.0008 (11)	0.0104 (10)
C27	0.0570 (15)	0.0603 (15)	0.0705 (18)	0.0097 (13)	−0.0064 (15)	0.0051 (13)
C28	0.075 (2)	0.0659 (17)	0.080 (2)	0.0235 (15)	0.0031 (18)	0.0023 (16)
C29	0.095 (2)	0.0579 (16)	0.069 (2)	0.0210 (16)	0.0033 (19)	−0.0031 (15)
C30	0.094 (2)	0.0654 (18)	0.067 (2)	0.0087 (16)	−0.0096 (17)	−0.0061 (15)
C31	0.0674 (18)	0.0601 (16)	0.0600 (17)	0.0098 (14)	−0.0075 (14)	−0.0014 (13)

### Geometric parameters (Å, °)

**Table:**

S1—C7	1.742 (2)	C16—C17	1.359 (5)
S1—C8	1.811 (3)	C16—H16	0.9300
S2—C4	1.752 (2)	C17—C18	1.357 (5)
S2—C20	1.818 (3)	C17—H17	0.9300
N1—C5	1.335 (3)	C18—C19	1.376 (4)
N1—N2	1.353 (3)	C18—H18	0.9300
N2—C3	1.341 (3)	C19—H19	0.9300
N2—C6	1.429 (3)	C20—C21	1.496 (4)
C3—C4	1.365 (3)	C20—C25	1.528 (3)
C3—H3	0.9300	C20—H20	0.9800
C4—C5	1.417 (3)	C21—C22	1.529 (4)
C5—C26	1.471 (3)	C21—H21A	0.9700
C6—C7	1.330 (3)	C21—H21B	0.9700
C6—C14	1.481 (3)	C22—C23	1.511 (4)
C7—H7	0.9300	C22—H22A	0.9700
C8—C9	1.506 (4)	C22—H22B	0.9700
C8—C13	1.522 (4)	C23—C24	1.504 (5)
C8—H8	0.9800	C23—H23A	0.9700
C9—C10	1.507 (5)	C23—H23B	0.9700
C9—H9A	0.9700	C24—C25	1.508 (4)
C9—H9B	0.9700	C24—H24A	0.9700
C10—C11	1.515 (5)	C24—H24B	0.9700
C10—H10A	0.9700	C25—H25A	0.9700
C10—H10B	0.9700	C25—H25B	0.9700
C11—C12	1.495 (5)	C26—C31	1.373 (4)
C11—H11A	0.9700	C26—C27	1.399 (4)
C11—H11B	0.9700	C27—C28	1.378 (4)
C12—C13	1.514 (5)	C27—H27	0.9300
C12—H12A	0.9700	C28—C29	1.350 (5)
C12—H12B	0.9700	C28—H28	0.9300
C13—H13A	0.9700	C29—C30	1.371 (5)
C13—H13B	0.9700	C29—H29	0.9300
C14—C19	1.374 (4)	C30—C31	1.379 (4)
C14—C15	1.385 (3)	C30—H30	0.9300
C15—C16	1.390 (4)	C31—H31	0.9300
C15—H15	0.9300		
			
C7—S1—C8	99.80 (12)	C15—C16—H16	119.7
C4—S2—C20	103.31 (11)	C18—C17—C16	119.8 (3)
C5—N1—N2	105.4 (2)	C18—C17—H17	120.1
C3—N2—N1	111.50 (19)	C16—C17—H17	120.1
C3—N2—C6	128.7 (2)	C17—C18—C19	120.8 (3)
N1—N2—C6	119.76 (19)	C17—C18—H18	119.6
N2—C3—C4	108.2 (2)	C19—C18—H18	119.6
N2—C3—H3	125.9	C14—C19—C18	120.3 (3)
C4—C3—H3	125.9	C14—C19—H19	119.8
C3—C4—C5	104.3 (2)	C18—C19—H19	119.8
C3—C4—S2	123.7 (2)	C21—C20—C25	110.3 (2)
C5—C4—S2	131.83 (19)	C21—C20—S2	114.20 (19)
N1—C5—C4	110.6 (2)	C25—C20—S2	106.29 (18)
N1—C5—C26	117.8 (2)	C21—C20—H20	108.6
C4—C5—C26	131.6 (2)	C25—C20—H20	108.6
C7—C6—N2	118.8 (2)	S2—C20—H20	108.6
C7—C6—C14	125.2 (2)	C20—C21—C22	110.9 (3)
N2—C6—C14	116.0 (2)	C20—C21—H21A	109.5
C6—C7—S1	124.6 (2)	C22—C21—H21A	109.5
C6—C7—H7	117.7	C20—C21—H21B	109.5
S1—C7—H7	117.7	C22—C21—H21B	109.5
C9—C8—C13	109.9 (2)	H21A—C21—H21B	108.0
C9—C8—S1	112.4 (2)	C23—C22—C21	111.6 (3)
C13—C8—S1	108.1 (2)	C23—C22—H22A	109.3
C9—C8—H8	108.8	C21—C22—H22A	109.3
C13—C8—H8	108.8	C23—C22—H22B	109.3
S1—C8—H8	108.8	C21—C22—H22B	109.3
C8—C9—C10	112.8 (3)	H22A—C22—H22B	108.0
C8—C9—H9A	109.0	C24—C23—C22	110.7 (3)
C10—C9—H9A	109.0	C24—C23—H23A	109.5
C8—C9—H9B	109.0	C22—C23—H23A	109.5
C10—C9—H9B	109.0	C24—C23—H23B	109.5
H9A—C9—H9B	107.8	C22—C23—H23B	109.5
C9—C10—C11	110.1 (3)	H23A—C23—H23B	108.1
C9—C10—H10A	109.6	C23—C24—C25	111.0 (3)
C11—C10—H10A	109.6	C23—C24—H24A	109.4
C9—C10—H10B	109.6	C25—C24—H24A	109.4
C11—C10—H10B	109.6	C23—C24—H24B	109.4
H10A—C10—H10B	108.1	C25—C24—H24B	109.4
C12—C11—C10	111.7 (3)	H24A—C24—H24B	108.0
C12—C11—H11A	109.3	C24—C25—C20	110.9 (2)
C10—C11—H11A	109.3	C24—C25—H25A	109.5
C12—C11—H11B	109.3	C20—C25—H25A	109.5
C10—C11—H11B	109.3	C24—C25—H25B	109.5
H11A—C11—H11B	107.9	C20—C25—H25B	109.5
C11—C12—C13	111.9 (3)	H25A—C25—H25B	108.1
C11—C12—H12A	109.2	C31—C26—C27	117.7 (2)
C13—C12—H12A	109.2	C31—C26—C5	119.2 (2)
C11—C12—H12B	109.2	C27—C26—C5	123.1 (2)
C13—C12—H12B	109.2	C28—C27—C26	119.7 (3)
H12A—C12—H12B	107.9	C28—C27—H27	120.2
C12—C13—C8	110.9 (3)	C26—C27—H27	120.2
C12—C13—H13A	109.5	C29—C28—C27	121.7 (3)
C8—C13—H13A	109.5	C29—C28—H28	119.1
C12—C13—H13B	109.5	C27—C28—H28	119.1
C8—C13—H13B	109.5	C28—C29—C30	119.4 (3)
H13A—C13—H13B	108.1	C28—C29—H29	120.3
C19—C14—C15	119.0 (2)	C30—C29—H29	120.3
C19—C14—C6	121.5 (2)	C29—C30—C31	119.8 (3)
C15—C14—C6	119.5 (2)	C29—C30—H30	120.1
C14—C15—C16	119.5 (3)	C31—C30—H30	120.1
C14—C15—H15	120.2	C26—C31—C30	121.7 (3)
C16—C15—H15	120.2	C26—C31—H31	119.2
C17—C16—C15	120.6 (3)	C30—C31—H31	119.2
C17—C16—H16	119.7		
			
C5—N1—N2—C3	1.2 (3)	C7—C6—C14—C15	−51.7 (4)
C5—N1—N2—C6	179.9 (2)	N2—C6—C14—C15	129.7 (2)
N1—N2—C3—C4	−1.5 (3)	C19—C14—C15—C16	−0.6 (4)
C6—N2—C3—C4	179.9 (2)	C6—C14—C15—C16	177.7 (2)
N2—C3—C4—C5	1.1 (3)	C14—C15—C16—C17	0.6 (4)
N2—C3—C4—S2	−175.90 (18)	C15—C16—C17—C18	0.1 (5)
C20—S2—C4—C3	−96.9 (2)	C16—C17—C18—C19	−0.8 (5)
C20—S2—C4—C5	86.9 (3)	C15—C14—C19—C18	−0.1 (4)
N2—N1—C5—C4	−0.4 (3)	C6—C14—C19—C18	−178.3 (3)
N2—N1—C5—C26	−178.1 (2)	C17—C18—C19—C14	0.8 (5)
C3—C4—C5—N1	−0.5 (3)	C4—S2—C20—C21	57.0 (2)
S2—C4—C5—N1	176.23 (19)	C4—S2—C20—C25	178.85 (19)
C3—C4—C5—C26	176.9 (3)	C25—C20—C21—C22	56.0 (4)
S2—C4—C5—C26	−6.4 (4)	S2—C20—C21—C22	175.7 (2)
C3—N2—C6—C7	164.4 (2)	C20—C21—C22—C23	−55.8 (4)
N1—N2—C6—C7	−14.1 (3)	C21—C22—C23—C24	55.3 (4)
C3—N2—C6—C14	−16.9 (3)	C22—C23—C24—C25	−56.3 (4)
N1—N2—C6—C14	164.5 (2)	C23—C24—C25—C20	57.3 (4)
N2—C6—C7—S1	177.14 (18)	C21—C20—C25—C24	−57.3 (3)
C14—C6—C7—S1	−1.4 (4)	S2—C20—C25—C24	178.4 (2)
C8—S1—C7—C6	−150.7 (2)	N1—C5—C26—C31	18.0 (3)
C7—S1—C8—C9	67.3 (2)	C4—C5—C26—C31	−159.2 (3)
C7—S1—C8—C13	−171.3 (2)	N1—C5—C26—C27	−160.5 (2)
C13—C8—C9—C10	56.4 (4)	C4—C5—C26—C27	22.3 (4)
S1—C8—C9—C10	176.9 (3)	C31—C26—C27—C28	2.3 (4)
C8—C9—C10—C11	−55.9 (5)	C5—C26—C27—C28	−179.1 (3)
C9—C10—C11—C12	54.5 (5)	C26—C27—C28—C29	−2.1 (5)
C10—C11—C12—C13	−55.3 (4)	C27—C28—C29—C30	0.5 (5)
C11—C12—C13—C8	55.5 (4)	C28—C29—C30—C31	0.9 (5)
C9—C8—C13—C12	−55.1 (3)	C27—C26—C31—C30	−1.0 (4)
S1—C8—C13—C12	−178.1 (2)	C5—C26—C31—C30	−179.6 (3)
C7—C6—C14—C19	126.5 (3)	C29—C30—C31—C26	−0.6 (5)
N2—C6—C14—C19	−52.1 (3)		

### Hydrogen-bond geometry (Å, °)

**Table:**

*D*—H···*A*	*D*—H	H···*A*	*D*···*A*	*D*—H···*A*
C7—H7···N1	0.93	2.40	2.760 (4)	103
C27—H27···S2	0.93	2.80	3.450 (4)	128
C31—H31···N1	0.93	2.46	2.786 (4)	101
